# The Clinical Laboratory as an Adjunct to Medicine*Read before the Brazos Valley Medical Association, at Caldwell, Texas, May 9, 1900.

**Published:** 1900-08

**Authors:** R. L. Jones

**Affiliations:** Cameron, Texas


For Texas Medical Journal.

### The Clinical Laboratory as an Adjunct to Medicine.*

BY R. L. JONES, M. D., CAMERON, TEXAS.

As medicine becomes a more and more exact science, its progress is marked in none of its branches more than in the all important one of diagnosis, and as methods and instruments of precision are rapidly taking the place of, or are being added to, the cruder methods of observation, this branch is taking its place in the front rank of scientific exactness.

---

*Read before the Brazos Valley Medical Association, at Caldwell, Texas, May 9, 1900.

It is not my intention to recall the many discoveries in pathology and bacteriology which have done so much to advance our art, but merely to briefly mention some of the laboratory methods of practical importance to the physician and surgeon.

To the practitioner of medicine the examination of the urine, further than the testing for albumen and sugar, is perhaps the most important. When albuminuria exists, it is important to know its exact amount, and whether this amount is increasing or decreasing daily under the treatment. The presence of albumen calls for an examination for tube casts, and if found, their number and variety will throw much light on the nature of the existing renal disease.

In obscure cases of pyuria, by a careful examination of the epithelial cells accompanying the pus, the seat of the lesion can sometimes be located; in the urethra, bladder, ureter, pelvis, parenchyma of the kidneys, as the case may be.

An estimation of the total amount of solids daily excreted throws much light on certain errors of metabolism.

Blood, in amounts too small to be detected in any other way, may be found with the microscope, and by a careful examination of the cells which accompany it, its origin may be approximately determined.

Of the micro-organisms to be found in the urine, the most important are the *bacillus tuberculosis* and the gonococcus of Neisser, Tuberculosis of the uro-genital tract may often be discovered and even located before a positive diagnosis can be otherwise made.

Examination of the feces, though less important, is sometimes necessary. In them may be found the ova of the intestinal worms, the amœba of tropic dysentery, the spirillum of cholera, and, though too late for use in diagnosis, the *bacillus typhosus.*

In the sputum, most important is the *bacillus tuberculosis.* It is true that it is claimed by some that we should diagnose phthisis pulmonalis long before it is revealed by the microscope, yet some of us might be in doubt, judging only from the physical signs and symptoms, and the finding of the specific bacillus is positive proof. Moreover, it serves to convince a patient of his condition and arouses him from his fancied security.

The chemical and microscopic examination of the vomitus is sometimes of value. In the differential diagnosis of gastric ulcer and gastric cancer, it will be remembered that in ulcer there is an excess of hydrochloric acid, while in cancer it is absent, and lactic acid is present.

The prsence of poisons may be detected by chemical examination of the contents of the stomach, which is chiefly of importance in medico-legal cases.

Much aid may be obtained in the examination of the blood; the various anemias may be differentiated and their progress watched, especially in chlorosis and chronic lead poisoning.

Finding of parasites in the blood is of less importance.

The *plasmodium malariæ* may be found, but it is seldom needed to diagnose malaria. The *filaria sanguinis hominis*, the cause of chyluria, is so rare in this country as to be unimportant.

Widol's test for typhoid, which may be classed with blood examinations, is one of the most important diagnostic discoveries of this decade. Its efficiency is impaired, however, by the fact that recent typhoid (within ten years), is not eliminated, and also, by the fact that considerable skill and care is necessary for its successful use.

To the surgeon the laboratory is still more important. A large part of the surgeon's work is in the treatment of tumors, and often the absolute diagnosis depends on the microscope.

To be able to say positively whether a neoplasm is malignant or benign, not only influences our prognosis, but decides whether or not operative procedures shall be instituted.

How often we are puzzled as to the nature of a tumor, whether it be of the breast, uterus, or elsewhere, when the microscope will completely clear up the case. The examination of the blood is also essential in many surgical cases. The estimate of the hæmaglobin yields important information after severe concealed hemorrhage, such as occurs in extra-uterine pregnancy, rupture of aneurysms or hemorrhage from the liver or spleen. In malignant disease the per cent. of hæmaglobin is far below normal, and, as after the hemorrhage attending the operation it never again reaches the normal per cent., it is of great importance in deciding for or against operation in advanced cases. In contemplated removal of the spleen, in the light of today, it is almost criminal to operate without first excluding leukemic spleen, which can only be done by a count of the corpuscles. It is almost equally true in cases of lymphatic leukemia, as a patient suffering from this disease could ill afford to lose a group of leukemic glands, as the condition would never recover from the loss.

In intra-abdominal conditions, the degree of leucocytosis throws light on whether or not the condition is inflammatory. This is therefore of special advantage in deciding whether an intestinal obstruction is simple, inflammatory, or malignant. If the blood is normal a non-inflammatory obstruction is certain, while a marked leucocytosis indicates an inflammatory cause, and leucocytosis with distortion of the red cells, lessened hemaglobin, accompanied by pus in the feces, indicates malignancy.

In conclusion, I will say a few words in regard to the preservation of specimens, where they are to be sent to a distant laboratory. Sputum will keep for several days, just as it is expectorated, but the bottle into which it is expectorated should be absolutely clean. Urine is best preserved by the addition of a few drops of chloroform. Pieces of tissue, such as glands, and fragments of tumors, are best preserved in alcohol, into which they should be placed immediately on removal from the body. The pieces should be small, about a third of an inch each way, and the amount of alcohol should be at least twenty-five times the volume of the tissue. The best method is to place them in absolute alcohol (which should contain not more than one-half of one per cent. of water), but if this can not be obtained, they should be put into a 70 per cent. solution, rather than into the ordinary 95 per cent., commonly found in drug stores and saloons. In either case the pathologist should be informed as to the strength of the preserving fluid, together with a short history of the case and its clinical features, in order that he may have an idea what to look for.

